# Seasonal Dynamics of Soil Microbial Biomass C and N along an Elevational Gradient on the Eastern Tibetan Plateau, China

**DOI:** 10.1371/journal.pone.0132443

**Published:** 2015-07-06

**Authors:** Xiaolin Gou, Bo Tan, Fuzhong Wu, Wanqin Yang, Zhengfeng Xu, Zhiping Li, Xitao Zhang

**Affiliations:** Key Laboratory of Ecological Forestry Engineering, Institute of Ecology & Forestry, Sichuan Agricultural University, No. 211, Huimin Road, Wenjiang District, Chengdu 611130, P. R. China; ENEA Casaccia Research Centre, ITALY

## Abstract

Little information is available on the seasonal response of soil microbial biomass to climate warming even though it is very sensitive to climate change. A two-year field experiment was conducted in the subalpine and alpine forests of the eastern Tibetan Plateau, China. The intact soil cores from 3,600 m site were incubated in three elevations (3,000 m, 3,300 m and 3,600 m) to simulate climate warming. Soil microbial biomass carbon (MBC) and nitrogen (MBN) were measured at different periods (early growing season [EG], late growing season [LG], onset of soil freezing period [OF], deep soil frozen period [DF] and soil thawing period [ET]) from May 2010 to August 2012. Average air temperature and soil temperature increased with the decrease of elevation during the experimental period. MBC and MBN showed a sharp decrease during the OF and ET in both organic layer and mineral layer at the three sites. Additionally, a relatively high MBC was observed during the DF. MBC and MBN in the soil organic layer decreased with the decrease of elevation but the opposite was true in the mineral soil layer. Warming had stronger effects on soil microbial biomass in the organic layer than in the mineral soil layer. The results indicated that future warming would alter soil microbial biomass and biogeochemical cycling in the forest ecosystems on the eastern Tibetan Plateau.

## Introduction

A growing body of evidence has demonstrated that climate change is occurring more intensely at higher elevations and latitudes in cold regions [1]. Soil microbes play critical roles in C and nutrient transformation in forest soils [2]. Soil microbial biomass not only lays their important roles as driving force in soil processes (e.g. N mineralization), but also acts as sensitive bio-indicator to ongoing climate change [3]. Generally, temperature is one of the main factors limiting the activity and reproduction of soil microbes in cold bioregions. However, studies of soil microbial biomass and its response to climate change have produced inconsistent results. Many studies observed that climate warming resulted in an increase in soil microbial biomass [4], but others declared soil microbial biomass decreased when temperature increasing [5]. Moreover, a few studies even documented that climatic warming did not affect microbial biomass [6].

These inconsistent observations may attribute to at least two related causes. On the one hand, various methods have been applied in experimental warming studies in cold bioregions. For example, either open top chambers or heating lamps have been used to stimulate climate warming [7, 8], but whether these findings can be reproduced under field conditions is uncertain. In contrast, temperature increases with change in elevational gradient in the field may provide a better way to mimic ongoing climate change [9, 10]. On the other hand, numerous studies have focused on the effects of long-term temperature changes on microbial biomass, but microbial characteristics are affected by changing temperatures in cold bioregions through at least three periods during winter as described below [11]. First, onset of the soil freezing period (OF), which is characterized by frequent freeze-thaw events as soil temperatures fall to the freezing point repeatedly until the first snowfall when the soil completely frozen. Second, deep soil frozen period (DF) which is characterized by soil temperatures remain below freezing point. Third, soil thawing period (ET) is also characterized by frequent freeze-thaw events as soil temperatures completely above the freezing point during early spring. These three periods might affect the response of soil microbial biomass to climatic warming in cold regions [12]. Moreover, the insulation of snow cover can prevent soil temperature from paralleling air temperature [13]. As a result, decreased in snow cover under climate warming will promote colder soil temperatures [1, 13], more frequent freeze-thaw cycles as well as decreased overall microbial activity [5, 14]. This may cause soil microbial biomass change with temperature increases in frost-free regions (the tropics) or seasons (growing season). Unfortunately, available studies on the subject have not adequately addressed this particular winter stage, making the relationship between soil microbial biomass and temperature unclear.

The alpine forests of Western China locate in the transitional area between the Qinghai–Tibet Plateau and the Sichuan Basin [15]. Soil in these forests consists of a thick organic layer but a thin mineral soil layer [16]. Previous studies have showed that the dynamics surrounding freezing and snowpack development and subsequent thawing often last about half a year [15]. Moreover, the magnitude of global warming on the Tibet Plateau is projected to be larger relative to other temperate regions at the same latitude [1]. The structure and activity of microbial community are significantly controlled by the seasonal freeze-thaw cycle in this region [11, 16, 17]. However, little attention has been paid to the responses of soil microbial biomass to climatic warming in this region.

In this study, we test the hypothesis that the increase in air temperature with the decrease of elevation can enhance soil microbial biomass in the entire year in alpine forest, and the increase of soil microbial biomass would differ between the freeze-thaw period and growing season. Therefore, a two-year field experiment was conducted in the eastern Tibetan Plateau, China. Intact soil cores sampled from 3600-m were transferred to 3300-m and 3000-m to simulate ongoing climatic warming. Soil microbial biomass of the organic soil layer and mineral soil layers were measured at different periods (early growing season, late growing season, onset of soil freezing period, deep soil frozen period and soil thawing period) from May 2010 to August 2012. The results could also be useful in explaining details of microbes and their related ecological processes in cold regions under climatic warming scenarios.

## Material and Methods

### Ethics Statement

The Institute of Ecological Forestry, Sichuan Agricultural University has had a permit from the Western Sichuan Forestry Bureau to conduct scientific experiments in the Bipenggou Nature Reserve since March 2006. The senescent fresh foliar litters collected for this study were only sampled at a very limited scale, and thus, had negligible effects on broader ecosystem functioning. Moreover, this research was carried out in compliance with the laws of People's Republic of China. The research did not involve measurements on humans or animals and no endangered or protected plant species was involved.

### Site description

This study was conducted in the Bipenggou Nature Reserve of Lixian County, Sichuan, China (102°53'–102°57'E, 31°14'–31°19'N, 2458-m to 4619-m a.s.l.). Annual mean temperature is 3.0 ± 0.5°C with maximum of 23.1 ± 1.1°C (July) and minimum of –18.0 ± 1.3°C (January), respectively. Mean annual precipitation ranges from 801 mm to 875 mm depending on elevations, and most of precipitation falls between May and August. Soil temperature goes down below 0°C and remains frozen during the whole cold snow season from late-November to mid-April [15].

Three sites were selected covering a 600 m vertical transition zone with elevations around 3,000 m (A1), 3,300 m (A2) and 3,600 m (A3) with similar topographic and environmental factors, such as slope, aspect and forest type. The forest is dominated by spruce and fir (*Abies faxoniana*), with dwarf bamboo (*Lonicera* spp.) and *Rubus corchorifolius* at the A1; by spruce, fir and birch (*Betula albosinensis*) with dwarf bamboo at the A2; and by fir and larch (*Larix mastersiana*) with a few azalea shrubs (*Rhododendron* spp.) and willow (*Salix paraplesia*) at the A3. Frequent freezing and thawing events occur before the soil freezes and after it thaws in these forests [17].

### Experimental design

Intact soil cores were sampled with PVC pipes (d = 11 cm, h = 20 cm) in an alpine primary forest at the A3 on May 24, 2010. Soil in the sampled forest is classified as Cambic Umbrisols [18] (IUSS Working Group WRB, 2007) with a ~11 cm deep organic matter layer. The basic chemical properties of soils are showed in Table 1. A total of 150 pipes were pushed into soil, and intact pipes were carefully excavated from soil. Both the top and bottom of pipe were sealed with nylon cloth to exclude foreign matter input. Fifty pipes were buried into soil in the sampled forest at the A3, and others pipes were respectively buried into soil at the A1 (50 pipes) and A2 (50 pipes).

**Table 1:** Basic chemical properties of soil in the soil organic layer (OL) and mineral soil layer (ML) of the fir forest.

Soil layer	pH	Soil bulk density (g.cm^-3^)	Organic C (g.kg^-1^)	Total N (g.kg^-1^)	Ammonium (mg.kg^-1^)	Nitrate (mg.kg^-1^)	Microbial biomass C (mg.kg^-1^)	Microbial biomass N (mg.kg^-1^)
OL	5.60 (0.80)^#^	1.05 (0.02)	150.1 (4.21)	9.70 (0.51)	22.84 (4.33)	143.20 (26.84)	647.35 (59.87)	76.51 (12.36)
ML	5.30 (0.90)	1.22 (0.14)	45.10 (1.03)	1.80 (0.12)	13.09 (2.51)	29.37 (7.86)	178.12 (37.62)	22.19 (3.65)

^#^ Data in parentheses are the standard deviation.

Abbreviations: OL, organic soil layer; ML, mineral soil layer.

To qualify the seasonal dynamics of soil microbial biomass C and N along an elevational gradient at different critical periods based on the field investigation and previous local data, a total of ten samplings were performed on (1) August 12, 2010 and August 19, 2011 for the early growing season period (EG), (2) October 17, 2010 and October 18, 2011 for the late growing season period (LG), (3) December 16, 2010 and December 28, 2011 for the onset of the soil freezing period (OF), (4) March 3, 2011 and March 7, 2012 for the deep soil frozen period (DF) and April 19, 2011 and April 28, 2012 for the soil thawing period (ET). The growing season (GS) includes the EG and LG periods, while the non-growing season (NGS) includes the OF, DF and ET periods. On each sampling date, five PVC pipes were randomly collected. Soils in the pipe were passed through a 2.5 mm sieve after the removal of visible debris and then kept in the refrigerator at 4°C within less than one week for microbial analysis.

Temperature of both air (1 m above forest floor) and soil (5 cm depth and 15 cm depth) were recorded by buried Thermochron iButton DS1923–F5 Recorders (Maxim Dallas Semiconductor Corp., USA) every 1 h in the three sites between May 24, 2010 and August 25, 2012. Soil gravimetrical water in all sites was routinely measured.

### Soil microbial biomass analyses

Analysis of soil microbial biomass C (MBC) and N (MBN) were extracted by 0.5 M K2SO4 and then determined followed by the chloroform fumigation extraction method with a conversion factor of 0.45 for MBC and 0.54 for MBN [19, 20]. Soil extractable organic C and total N in these fumigated and unfumigated extracts were also determined by the dichromate oxidation-ferrous sulphate titration and semi-micro Kjeldahl method, respectively [20, 21]

### Statistical analyses

All variables were subjected to repeated measures ANOVA and significant differences between treatment means for each variable were compared by the Tukey’s HSD post-hoc test or the Student’s independent-sample t-test at P < 0.05. All statistical analyses were performed using 18.0 SPSS software package for Windows (SPSS Inc., IL, USA).

## Results

### Temperature

**Fig 1:**
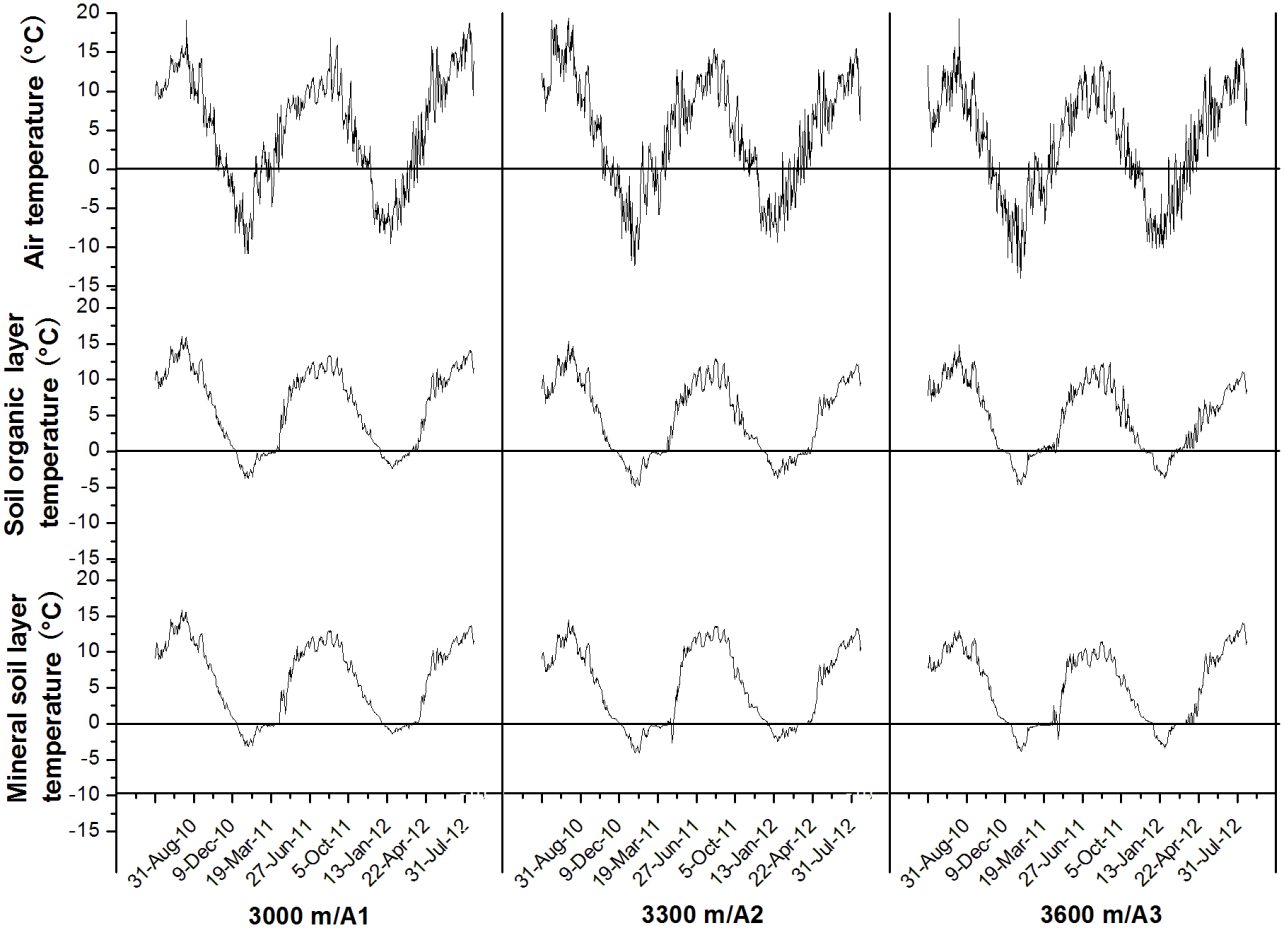
Daily mean air temperature and soil temperature at organic soil layer and mineral soil layer at different elevation of subalpine and alpine forests in the eastern Qinghai-Tibetan Plateau.

**Fig 2:**
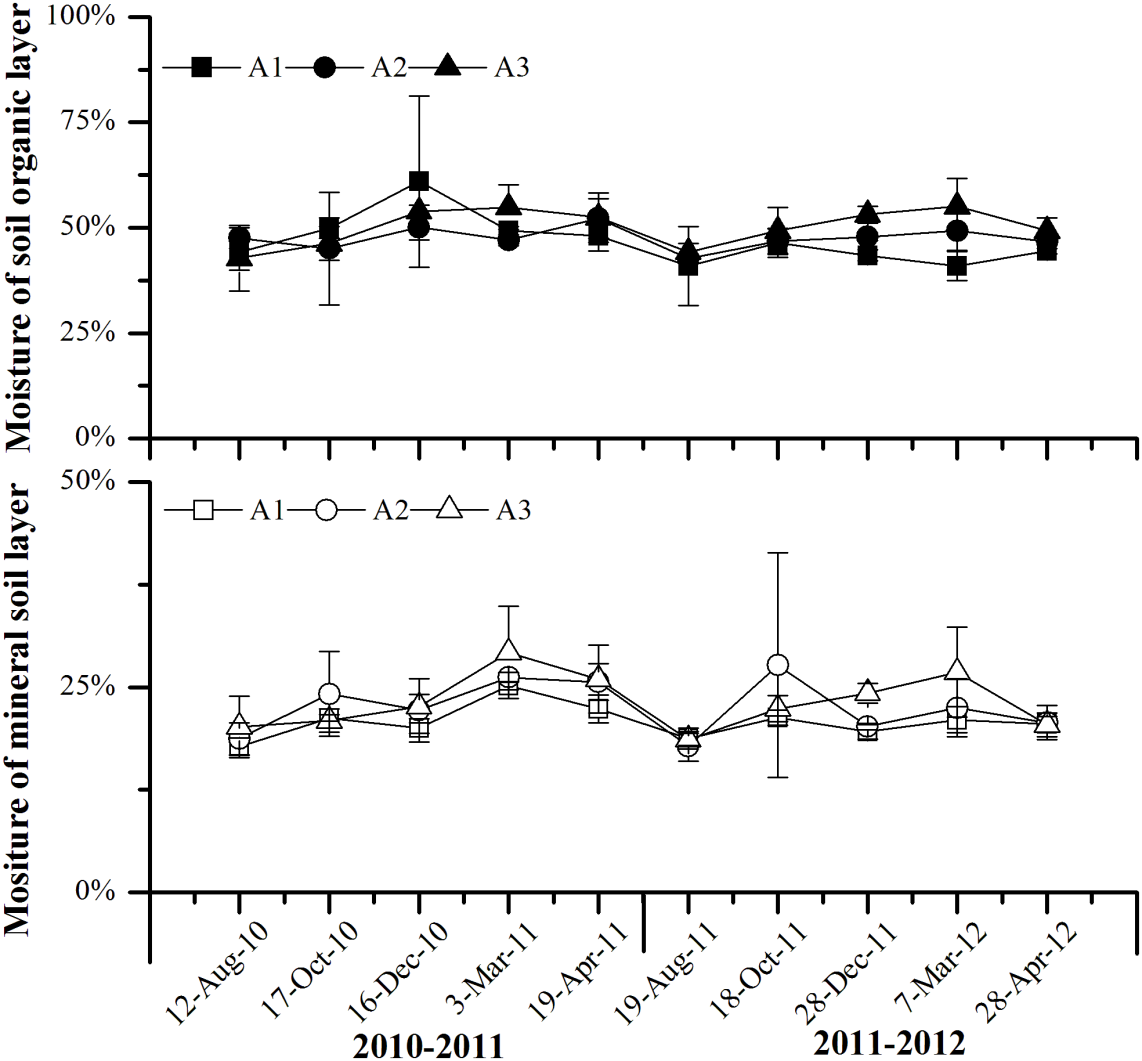
Effects of experimental warming on the dynamics of soil gravimetrical water at different elevation of subalpine and alpine forests in the eastern Qinghai-Tibetan Plateau. Error bars indicate standard error. *P < 0.05. *n* = 5. Abbreviations: OL, organic soil layer; ML, mineral soil layer; A1, 3000 m; A2 3300 m; A3, 3600 m.

Obviously temperature fluctuations were recorded during the study period at the three sites (Fig 1). Minimum and Average air temperature and soil temperature increased with the decrease of elevation during the study period (Table 2). As compared with air temperatures at the 3600 m, average air temperatures at the 3000 m and at the 3300 m were 2.01°C and 1.49°C higher, respectively. Moreover, mean soil temperature in the organic soil layer (OL) and mineral soil layer (ML) at the 3000 m was 1.49°C and 1.09°C higher than that at the 3600 m, respectively. However, there was little difference in mean soil temperature in both the OL and ML between the 3300 m and 3600 m. Soil gravimetrical water at the three sites showed little change during most of during the study period (Fig 2).

**Table 2:** Minimum, maximum and averaged air temperature and soil temperature (5 cm depth and 10 cm depth) in the fir forest at different altitudes.

Altitudes	Minimum (°C)			Maximum (°C)			Averaged (°C)		
	air	5 cm	15 cm	air	5 cm	15 cm	air	5 cm	15 cm
3600 m	-13.96a (0.18)^#^	-4.64a(0.34)	-3.90a(0.28)	19.29a (0.22)	14.89a (0.19)	14.06ab (0.36)	3.32a (0.27)	4.76a (0.32)	5.14a (0.28)
3300 m	-12.31b (0.20)	-4.94a(0.32)	-4.07a(0.31)	19.38a (0.33)	15.31b (0.21)	14.42a (0.25)	4.81b (0.19)	4.71a (0.22)	5.27a (0.25)
3000 m	-10.85c (0.21)	-3.79b(0.24)	-3.21b(0.27)	19.16a (0.24)	15.99c(0.23)	15.81b(0.26)	5.27c (0.23)	6.25b (0.41)	6.23b (0.31)

^#^ Data in parentheses are the standard deviation.

Values within a column followed by the same lower case letters are not significantly different at *P* < 0.05.

### Microbial biomass carbon (MBC)

**Fig 3:**
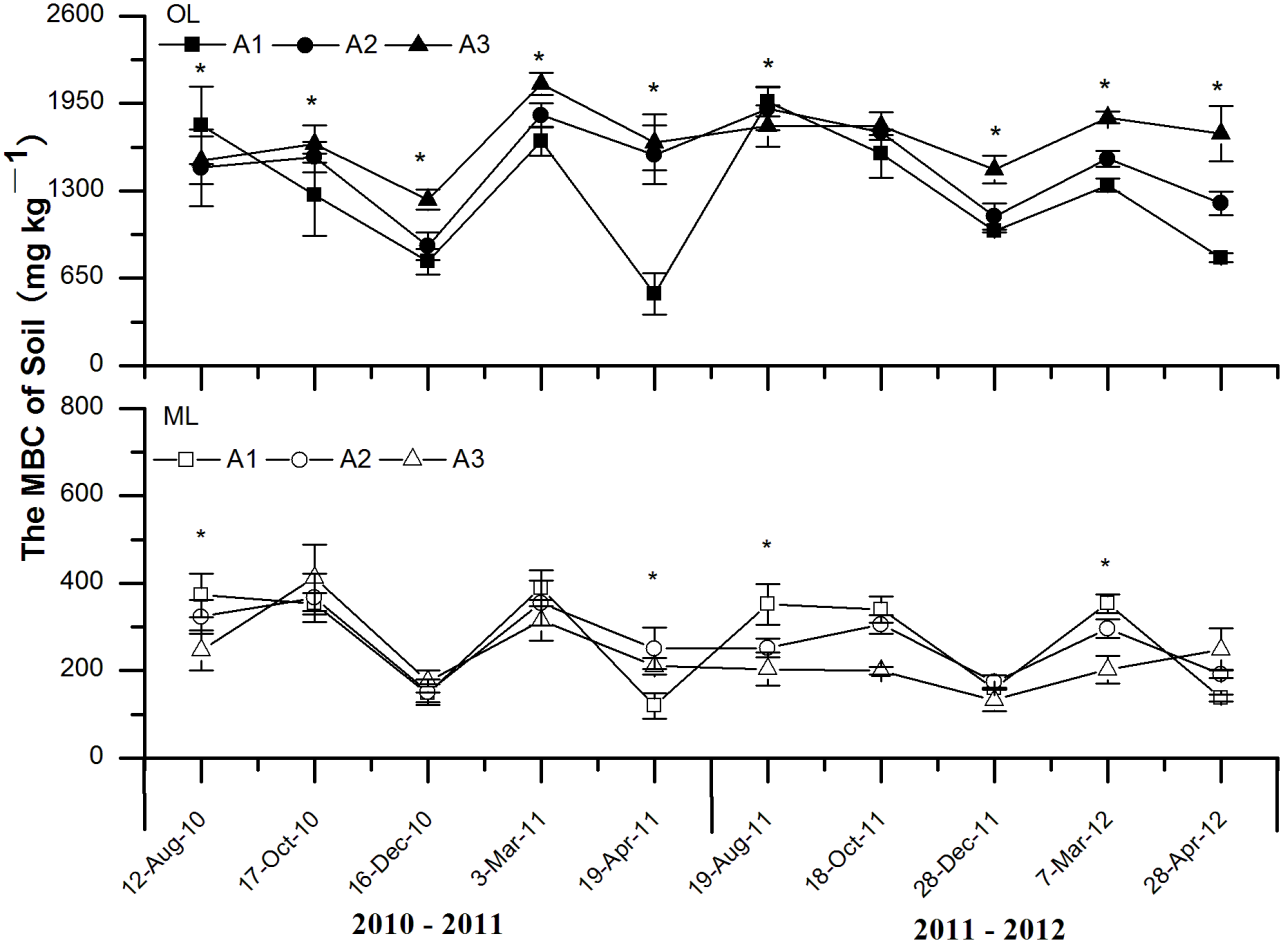
Effects of experimental warming on the dynamics of soil microbial biomass C (MBC) at different elevation of subalpine and alpine forests in the eastern Qinghai-Tibetan Plateau. Error bars indicate standard error. *P < 0.05. *n* = 5. Abbreviations: OL, organic soil layer; ML, mineral soil layer; A1, 3000 m; A2 3300 m; A3, 3600 m.

MBC in both the OL and ML displayed similar patterns with temperature fluctuations at the three elevations in our two year study (Fig 3). MBC in the two layers sharply decreased during the onset of soil freezing (OF) and soil thawing periods (ET), but a relatively high MBC were measured during the deep soil frozen period (DF). Altitude had significant effects on soil MBC in both the OL and ML during the study periods except for in the OL during the late growing (LG) period in the second year. MBC in the OL decreased with the decrease of elevation except for during the early growing period (EG). Few changes of MBC in the OL of soil were observed with the changes of soil temperature during the growing season (GS). In addition, altitude, sampling date, soil layer and their interaction all showed significant effects on MBC (Table 3).

**Table 3:** Results of repeated measures ANOVA showing the *P* values for responses of soil microbial biomass C (MBC) and N (MBN), and ratio of MBC to MBN to altitude (A), layer (L) sampling date (LT) and sampling dates (D).

Factor	MBC			MBN			MBC:MBN		
	*P*	*df*	F	*P*	*df*	F	*P*	*df*	F
A	<0.001	2	58.37	<0.001	2	73.03	0.079	2	4.69
D	<0.001	4	142.84	<0.001	4	164.56	<0.011	4	8.76
L	<0.001	1	6278.13	<0.001	1	5590.94	<0.001	1	3876.18
A×D	0.008	8	28.41	<0.001	8	9.77	<0.007	8	5.80
A×L	<0.001	2	70.81	<0.001	2	104.17	<0.001	2	8.64
D×L	0.017	4	52.93	<0.001	4	111.74	<0.001	4	46.19
A×L×D	<0.001	8	14.31	<0.001	8	11.90	<0.015	8	6.96

### Microbial biomass nitrogen (MBN)

**Fig 4:**
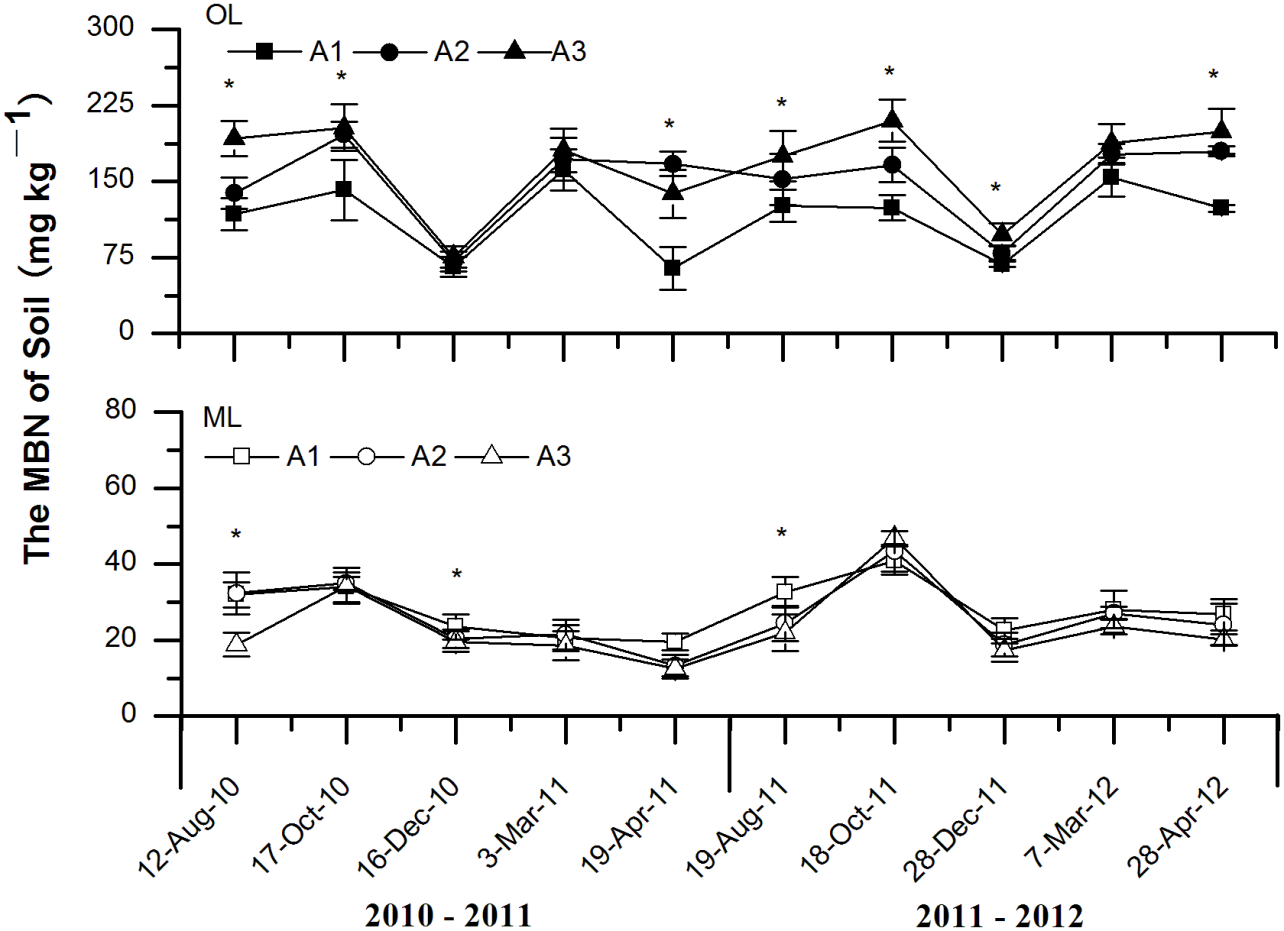
Effects of experimental warming on the dynamics of soil microbial biomass N (MBN) at different elevations of subalpine and alpine forests in the eastern Qinghai-Tibetan Plateau. Error bars indicate standard error. *P < 0.05. *n* = 5. Abbreviations: OL, organic soil layer; ML, mineral soil layer; A1, 3000 m; A2 3300 m; A3, 3600 m.

MBN showed a sharp decrease dynamic during the OF and ET in both the OL and ML at the three sites (Fig 4), but a relatively high MBC were also measured during the DF. Altitude had significant effects on soil MBN in the OL during the study periods except for the OF in the first year and DF in the second year. Moreover, altitude had significant effects on MBN in the ML during the EG period in both the two study years. MBN in the OL decreased with the decrease of elevation, but that in the ML increased with the decrease of elevation. In addition, altitude, sampling date, soil layer and their interaction all showed significant effects on MBC (Table 3).

### MBC/MBN

**Fig 5:**
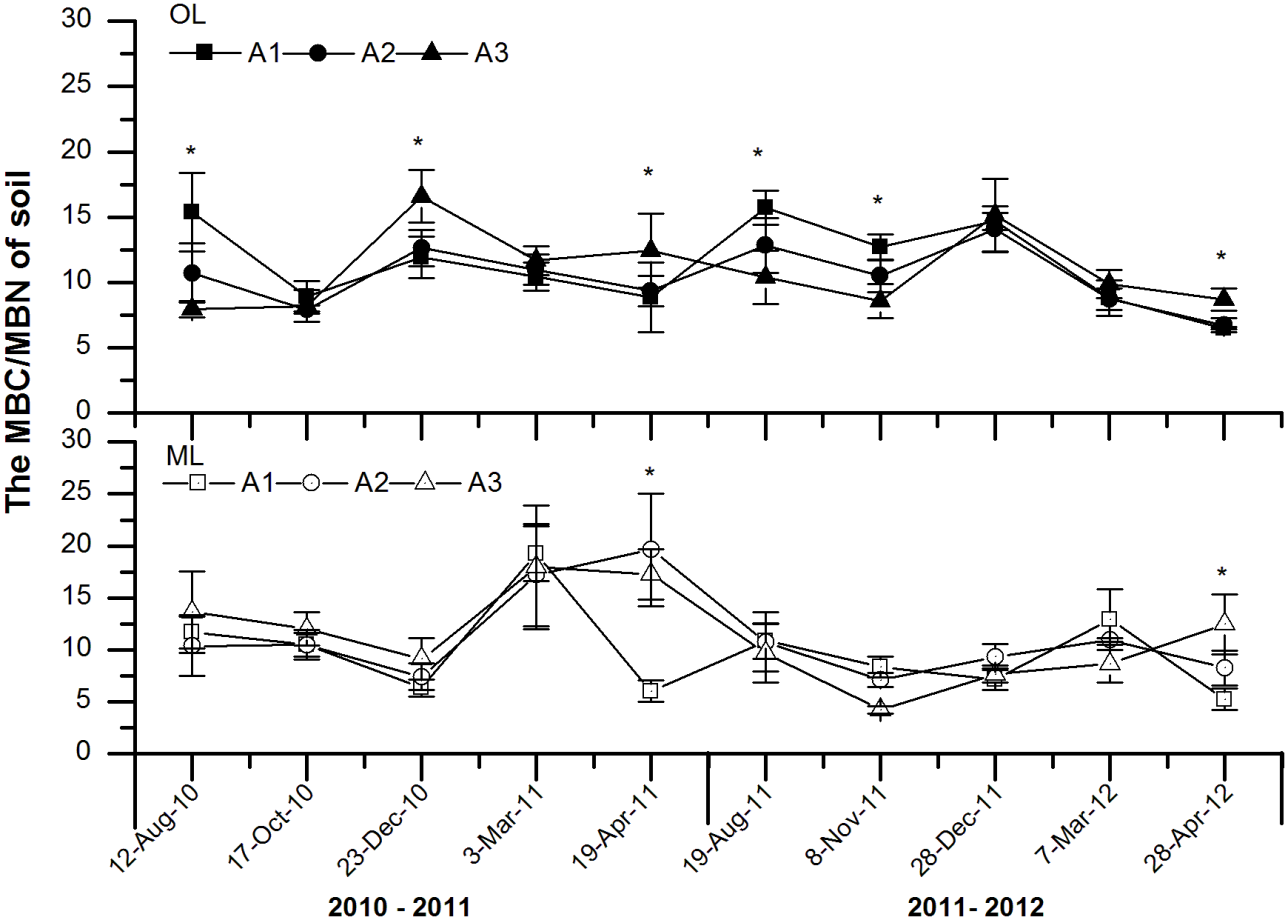
Effects of experimental warming on the ratios of soil microbial biomass C to N (MBC/MBN) at different elevations of subalpine and alpine forests in the eastern Qinghai-Tibetan Plateau. Error bars indicate standard error. *P < 0.05. *n* = 5. Abbreviations: OL, organic soil layer; ML, mineral soil layer; A1, 3000 m; A2 3300 m; A3, 3600 m.

During the two year study, the ratio of MBC to MBN in the OL slightly decreased during the LG, and that exhibited an obvious decrease between the OF and ET (Fig 5). However, the ratio of MBC to MBN in the ML showed a significant decrease dynamic during the OF in the first year and an obvious decrease dynamic during the LG in the second year. Moreover, the ratio of MBC to MBN in the soil OL increased with the decrease of elevation in the growing season (GS), but which decreased during the non-growing season (NGS) in two years study. In addition, altitude showed insignificant effects on the ratio of MBC to MBN (Table 3).

## Discussion

As we expected, average air temperature increased with the decrease of elevation during the study period. Air temperatures were 2.01°C higher at the 3000 m and 1.49°C higher at the 3300 m as compared with that at the 3600 m, respectively. Although the change of soil temperature did not exhibit a linear relationship with that of air temperature, the minimal and averaged soil temperatures were lower at the 3000 m than these at the 3600 m (Table 2), indicating an elevational gradient effect on soil temperature. Moreover, low altitude also resulted in relatively mild freezing events during winter, and the date of soil freezing and melting started earlier at the 3000 m than at the 3600 m (Fig 1). Indeed, the changes in soil freezing and subsequent thawing along the elevational gradient in winter might have affected dynamics of soil microbial biomass C (MBC) and N (MBN) in alpine forest in various ways [22]. In addition, soil gravimetrical water at the three sites showed little change during most of during the study period indicated that the change in precipitation along the elevational gradient in these sites might have little influence on dynamics of MBC and MBN.

The hypothesis that the increase in air temperature with the decrease of elevation can enhance soil microbial biomass in the entire year in alpine forest was only partly demonstrated here. In consistent with the result of Koch et al [23], our study showed that an increase in average microbial biomass in the mineral soil layer (ML) with the decrease of elevation, and *vice versa* in the organic soil layer (OL). During most of the sampling dates, MBC and MBN in the OL decreased with the decrease of elevation. This could be attributed to the protective effects of snow cover in winter and the relatively rich substrate input from snowmelt before the beginning of the growing season [15, 17, 24, 25]. On the other hand, soil microbial biomass did not always show similar patterns with the changes of soil temperature at different critical periods, especially for the winter. Studies in the arctic tundra and alpine ecosystem have demonstrated that soil microbial biomass often kept at an annual peak value during the winter [25]. This was also demonstrated in our study since a relatively high MBC and MBN were measured during the deep soil frozen period (DF). Moreover, the change of soil microbial biomass along the elevational gradient differed between the freeze-thaw period and growing season. There were several possible underlying mechanisms resulting in such changes. First, soil freezing and freeze-thaw cycle might damage soil microbes [26]. Subsequently, nutrients released from the cells of the senescing microbes could be utilized by surviving microbes. Second, increased freezing and thawing cycles could often exert greater damage on soil organisms [6, 7]. Third, during frozen soil thawing at the end of winter, increased temperature and abundant substrate input can significantly promote the growth of soil microbes [3, 5, 22], inducing higher soil microbial biomass during the early growing period (EG). Therefore, the partial immobilization of nutrients as microbial biomass might represent the immediate input of available nutrients into these forest soils for plant growth in the EG [2, 25]. In addition, soil microbial biomass in the OL showed greater response to experimental winter warming compared with the ML. Organic matter were much greater in the OL of alpine forests on the Tibetan Plateau relative to the ML. Thus, future warming could cause more significant effects on soil carbon and nitrogen processes in the OL of the studied forest ecosystems.

In general, soil temperature during the non-growing season (NGS) was colder than during the growing season (GS). Microbes grew better during the growing season (GS) with a warmer environment. However, the shortage of available carbon and nitrogen could limit the growth of soil microbial biomass during the late growing period (LG) because of that most of the available nutrients had been consumed by microbes during the early growing period (EG). Xu et al [27] found that warming enhanced soil respiration in the Tibetan Plateau. Schlesinger and Andrews [28] observed half of the respiration of a year occurred during the decomposition of soil organic matter. Therefore, MBN and MBC decreased in the OL because microbes consumed much of the available carbon and nitrogen. Moreover, soil freeze-thaw cycles can directly kill soil microbes. Warmer air promotes snowmelt, inducing an increase in the number of freeze-thaw cycles [29, 30], so that microbes were killed by serious environmental change at lower elevation in the OL [31]. In comparison with the OL, the ML faced fewer environmental changes because of its deeper depth, so that the ML could maintain a relatively stable condition for the growth of microbes. Moreover, nutrient leaching from the OL could also contribute to the increase of microbial biomass in the ML. The results also suggested that larger variations in soil temperature limited soil microbial activity and growth, which has significant implication for understanding soil ecological processes of alpine forests on the eastern Tibetan Plateau under global warming scenarios.

## Conclusions

In conclusion, the simulation of a climate warming can reduce soil microbial biomass in the organic soil layer (OL), but enhance it in the mineral soil layer (ML) in this alpine fir forest. MBC and MBN showed a sharp decrease dynamic during the onset of freezing and soil thawing periods in both the OL and ML at the three sites, but a relatively high MBC were also detected during the deep soil frozen period. Simulated warming had stronger effects on microbial biomass in the OL than that in the ML. These results indicate that change in soil temperature under global warming scenarios will alter soil microbial biomass and hence element biogeochemical cycling in alpine forest ecosystems.
